# Safety and efficacy of regional citrate anticoagulation in continuous blood purification treatment of patients with multiple organ dysfunction syndrome

**DOI:** 10.1590/1414-431X20176378

**Published:** 2017-11-17

**Authors:** B. Tuerdi, L. Zuo, H. Sun, K. Wang, Z. Wang, G. Li

**Affiliations:** 1 Respiratory Intensive Care Units, the First Affiliated Hospital of Xinjiang Medical University, Urumqi, Xinjiang, China; 2 Intensive Care Units, Branch of the First Affiliated Hospital of Xinjiang Medical University, Changji, Xinjiang, China

**Keywords:** Multiple organ dysfunction syndrome, Continuous blood purification, Blood coagulation function, Sodium citrate

## Abstract

The aim of this study was to discuss the safety and efficacy of regional citrate anticoagulation (RCA) on continuous blood purification (CBP) during the treatment of multiple organ dysfunction syndrome (MODS). Thirty-five patients with MODS were divided into two groups: the local citrate anticoagulation (RCA) group, and the heparin-free blood purification (hfBP) group. The MODS severity was assessed according to Marshall’s MODS score criteria. Blood coagulation indicators, blood pressure, filter lifespan, filter replacement frequency, anticoagulation indicators, and main metabolic and electrolyte indicators were analyzed and compared between RCA and hfBP groups. RCA resulted in lower blood pressure than hfBP. The filter efficacy in RCA treatment was longer than in the hfBP group. The blood clearance of creatine, blood urea nitrogen and uric acid was better in the RCA group. RCA also led to higher pH than hfBP. Neither treatment resulted in severe bleeding events. In addition, MODS score was positively correlated with prothrombin time and activated partial thromboplastin time but negatively correlated with platelet concentration. RCA is a safer and more effective method in CBP treatment; however, it could also lead to low blood pressure and blood alkalosis.

## Introduction

Multiple organ dysfunction syndrome (MODS) is a common critical illness, referring to the occurrence of simultaneous or sequential organ or system dysfunction 24 h after severe infection, wound shock, major surgery, severe pancreatitis, cardio-pulmonary resuscitation or other protopathies (1). It is generally believed that MODS is attributed to uncontrolled inflammatory mediators release, usually accompanied by inflammatory and metabolic disorders (2). Despite MODS pathogenesis being widely acknowledged and various therapies being introduced, the mortality of MODS is still much higher than 40%, ranking first among all critical illness-caused deaths (3,4).

Continuous blood purification (CBP), also named continuous renal replacement therapy, is a technology used to eliminate blood metabolites slowly and continually. As a new technology, CBP has already been applied in non-kidney disease treatments and has shown unique advantages when treating severe acute pancreatitis, systemic inflammatory response syndrome, sepsis, acute respiratory distress syndrome and MODS, becoming the main measure used to rescue critically ill patients (5). CBP is characterized by its hemodynamic stability, high metabolites clearance rate, efficiency of inflammatory mediator elimination and provision of nutritional support (6). In recent years, CBP has been widely applied to salvage and treat severely ill patients with remarkable therapeutic efficacy. CBP has been extensively applied in intensive care units in China, and its use on MODS treatment has been fully acknowledged (7). However, CBP needs continuous anticoagulation. Patients undergoing CBP are usually accompanied by active bleeding or a high risk of bleeding. The disseminated intravascular coagulation, triggered by severe wounds, infection, sepsis and shock, needs to be controlled (8). Insufficient anticoagulation (frequent coagulation) can suspend the treatment, and raise the clinical costs as well. On the other hand, over-anticoagulation may result in bleeding, or even life-threatening problems (9).

Therefore, the conduct of a safe and effective anticoagulation during CBP treatment is paramount. Although there are various anticoagulants available, none is omnipotent, given the variety of patients’ physical conditions (10). For instance, the commonly used heparin may result in severe bleeding; low-molecular-weight heparin needs a high water-flow rate and the flow increases the volume load, which cannot be tolerated by hemodynamically instable patients; trisodium citrate is not consistent, and the technical aspect of its use is rather complicated; and regional citrate anticoagulation (RCA) could lead to low blood pressure of patients. The safety and efficacy of various anticoagulants need to be evaluated to provide useful information for clinicians when choosing the appropriate anticoagulants for their patients. On the other hand, heparin-free blood purification (hfBP) combined with molecular adsorbent recirculating system (MARS) showed a better non-bleeding outcome. In addition, heparin-free-MARS purification did not compromise the circuit function and longevity (11). A study on unfractionated heparin in 61 MARS treatment sessions in 33 patients showed that the platelet count was reduced but the platelet function remained unchanged (12). Heparin-free purification was also required for patients without disseminated intravascular coagulation, who demonstrated some coagulation abnormalities and thrombocytopenia during coupled plasma filtration adsorption (CFPA) purification therapy (13).

In this study, RCA and hfBP were compared in terms of the anticoagulation safety and efficacy during CBP treatment of MODS patients. This study may provide evidence that RCA could be a better choice for MODS CBP treatments.

## Material and Methods

### Patients

Thirty-five patients (25 males and 10 females) with MODS who came to the hospital for CBP during June 2009 and September 2010 were enrolled in this study. The MODS of the 35 patients was caused by trauma (n=8), septic shock (n=10), severe acute pancreatitis (n=7), pregnancy-induced hypertension (n=3), postpartum hemorrhage (n=2), hepatic failure (n=1), open-heart surgery (n=3) and abdominal aortic aneurysm (n=1). All the included participants who agreed with this study were randomly assigned to RCA blood purification (RCA group, n=17) or the hfBP group (n=18). Written informed consent and ethics approval were signed by the patients’ legal representatives.

### Inclusion and exclusion criteria

Patients with MODS who needed CBP and met the MODS diagnosis criteria (the Marshall 1995 MODS score criteria, shown in Supplementary Table S1) were included (14,15). Those with MODS caused by hematologic system disorders or severe thrombocytopenia were excluded.

### Intervention

Diapact polysulfone HI PS 18 filter (B. Braun, Germany) and circuit tube were used to establish the extracorporeal circuit system. The patients in both groups were treated with continuous veno-venous hemofiltration (CVVH). Pre-dilution mode was used and the replacement fluid speed was 3000–4000 mL/h. The blood flow was kept above 150 mL/min. Ionized calcium (iCa) levels were measured 1 h after the start of CVVH and every 6 h thereafter.

Anticoagulant citrate dextrose A (ACD-A) solution (600 mL, purchased from Nigale Corporation, China) was used as the replacement fluid in the RCA group. The ACD-A infusion rate was 2–2.5% of blood flow rate, which was >150 mL/min. During this procedure, the concentration of main electrolytes reached the final 140 mmol/L of Na^+^, 105 mmol/L of Cl^-^, 0 mmol of Ca^2+^, 0.94 of Mg^2+^, 11.1 mmol/L of glucose, 21 mmol/L of HCO_3_^-^ and 4.6 mmol/L of citrate. Bicarbonate substitution fluid was used as the hfBP solution with 140 mmol/L of Na^+^, 110 mmol/L of Cl^-^, 1.5 mmol/L of Ca^2+^, 0.94 mmol/L of Mg^2+^, 10.5 mmol/L of glucose and 35 mmol/L of HCO_3_^-^.

### Adverse reactions

No reverse reactions were seen in either group. The evaluated reverse reactions include paresthesia, twitching movement, fever and bleeding complications.

### Study endpoints

Our study endpoints were efficacy and safety. Safety was defined as the lack of adverse events such as hemorrhage complications including digestive tract, respiratory tract, incision, oral mucosa and derma hemorrhage before and after treatment. The duration of the first filter and the replacement frequency of filters were assessed. Major bleeding was defined as the presence of severe bleeding with spontaneous blood pressure drop. Metabolic alkalosis was defined as pH>7.5. Hypocalcemia was defined as ionized calcium level <1.1 mmol/L.

### Data collection

The severity of MODS was scored using MODS score system before and after the treatment in the two groups. The Glasgow Coma Scale was calculated by a nurse who did not participate the study. The hemorrhage complications were assessed by a physician who was not involved in this study.

Coagulation indicators including prothrombin time (PT), PT activity, activated partial thromboplastin time (APTT), international normalized ratio (INR), fibrinogen (FIB) and platelet concentrations were recorded before and 24 h after CBP. Besides, the pressure of artery (PA), the pressure before hemofilter (PBE), the pressure of vein (PV), transmembrane pressure (TMP), the duration of filter, and the filter replacement frequency were also recorded by a physician. The pH and iCa levels of all participants were monitored four times every day. Electrolytes and platelet counts were measured daily. The level of sodium was also monitored to detect RCA-related electrolyte derangements.

### Statistical analyses

Data are reported as means±SD. Data were analyzed using the SPSS 20.0 package (IBM, USA). One-way ANOVA was used to compare differences of the indicators among different MODS groups before treatment. Parameters between the RCA and hfBP groups were compared using the *t*-test. Pre-treatment and post-treatment assessments were compared using the *t*-test. Bartlett sphericity test with or without Greenhouse-Geisser correction was performed to compare inter-group or intra-group data at different time points. Statistical significance was determined if P values were less than 0.05.

## Results

### Clinical characteristics of participants at the initiation of blood purification

Generally, there were no notable differences in the tested indicators between the RCA and hfBP groups (Table 1).

**Table 1.** Baseline characteristics of patients with multiple organ dysfunction syndrome (MODS) at the initiation of blood purification.

Variable	RCA group	Heparin-free group	t/χ2	P value
Age (years)	52.75±21.44	50.50±15.71	0.29	0.77
Gender (%)				
Male	70.6	61.1	0.35	0.41
Female	29.4	38.9		
MODS score	8.00±3.33	9.39±2.25	-1.37	0.18
PT (s)	16.86±3.82	17.79±2.44	-0.82	0.42
PT activity (%)	61.87±35.57	49.18±11.53	1.2	0.25
APTT (s)	54.40±53.76	31.98±4.23	1.44	0.18
INR	1.41±0.34	1.54±0.22	-1.22	0.23
FIB (g/L)	5.29±1.65	6.16±2.58	-1.03	0.31
Platlet concentration (109/L)	116.17±74.25	107.80±106.09	0.24	0.82
Sodium concentration (mmol/L)	134.92±5.96	137.67±6.52	-1.17	0.25
Calcium concentration (mmol/L)	1.98±1.10	1.97±0.26	0.58	0.97
BUN concentration (mmol/L)	25.69±22.08	27.87±15.35	-0.32	0.75
Creatine concentration (mmol/L)	423.00±472.64	439.67±219.31	-0.11	0.91
UAC Concentration (mmol/L)	338.67±204.91	365.94±189.66	-0.37	0.71

Data are reported as means±SD or number and percentage. RCA: regional citrate anticoagulation; PT: prothrombin time; APTT: activated partial thromboplastin time; INR: international normalized ratio; FIB: fibrinogen; BUN: blood urea nitrogen; UAC: uric acid. Statistical analysis was performed with the *t*-test or chi-square test.

### Blood coagulation indicators

In both groups, the coagulation indicators did not differ significantly before and after treatment. There was also no significant difference between the RCA post-treatment outcomes and the hfBP post-treatment outcomes (Table 2).

**Table 2.** Coagulation indicators at baseline and after continuous blood purification within regional citrate anticoagulation (RCA) or heparin-free blood purification (hfBP) groups.

	PT (s)	PT activity (%)	APTT (s)	INR	FIB (g/L)	Platelet concentration (10^9^/L)
RCA group						
Before treatment	16.86±3.82	61.87±35.57	54.40±53.76	1.14±0.34	5.29±1.65	116.17±74.25
After treatment	17.43±2.65	48.58±11.88	33.07±5.37	1.46±0.23	4.41±1.80	119.83±62.72
*t*	-0.49	1.43	1.41	-0.45	1.34	-0.19
P value	0.66	0.18	0.19	0.66	0.21	0.85
hfBP group						
Before treatment	17.79±2.44	49.18±11.53	31.98±4.23	1.54±0.22	6.16±2.58	107.80±106.09
After treatment	17.71±3.73	50.32±9.95	34.56±8.15	1.55±0.48	5.65±2.24	102.64±112.50
*t*	0.78	-0.38	-1.8	-0.14	1.1	0.78
P value	0.94	0.71	0.09	0.89	0.29	0.45

Data are reported as means±SD. PT: prothrombin time; APTT: activated partial thromboplastin time; INR: international normalized ratio; FIB: fibrinogen. Statistical analysis was performed with the *t*-test.

### Hemorrhage events and blood pressure comparison

No patients in either group showed hemorrhage symptoms (Table 3). Within each group, PA did not differ significantly between different time points, whereas PBE, PV and TMP altered substantially as the anticoagulation treatment time advanced. Except for TMP, the remaining tested indicators were significantly higher in the hfBP group than in the RCA group. RCA resulted in lower blood pressure than hfBP treatment, indicating that RCA might be a risk factor for bleeding.

**Table 3.** Anticoagulation efficacy of regional citrate anticoagulation (RCA) and heparin-free blood purification (hfBP) treatments.

Group	0 h	2 h	4 h	6 h	8 h	10 h	12 h	F value		P value	
								Inter-class	Intra-class	Inter-class	Intra-class
PA											
RCA	53.42±17.63	49±18.21	47.5±16.34	47.08±17.29	45.25±11.59	48.33±13.51	50.25±20.56	21.04	0.69	0.00	0.59
hfBP	83.50±40.31	74.11±29.81	86.56±37.91	83.06±29.32	80.83±26	75.61±21	88.89±37.86				
PBE											
RCA	99.08±35.08	106.08±27.17	116.58±23.71	112.25±23.25	112.92±23.99	121.92±47.97	141.83±83.95	16.09	6.46	0.00	0.00
hfBP	147.39±46.31	146.78±36.07	163.33±71.02	173.33±58.57	199.11±74.33	228.11±106.7	239.5±117.96				
PV											
RCA	43.17±19.31	43±14.69	49.58±16.21	47.42±15.58	47.5±15.83	90.67±173.77	59.33±37	8.00	3.73	0.00	0.04
hfBP	71.17±29.82	73±25.56	76.67±28.17	79.72±35.66	103.72±63.42	121.33±80.81	128.89±91.77				
TMP											
RCA	40.67±14.44	69.17±26.61	75.25±14.12	80.92±17	84.08±15.55	92.42±43.72	117.08±89.33	0.78	15.86	0.39	0.00
hfBP	46.39±32.89	60.22±20.6	69.5±26.01	97.89±56.44	105.17±66.77	122.78±69.65	135.89±72.99				

Data are reported as means±SD. PA: pressure of artery; PBE: pressure before hemofilter; PV: pressure of vein; TMP: transmembrane pressure. Statistical analysis was performed with one-way ANOVA.

### Lifespan and replacement frequency of the filter

The filter lifespan of the RCA group was approximately 29.8 h, whereas that of the hfBP group was about 12.5 h and this difference was significant. The replacement frequency of the RCA group (5.90%) was substantially smaller than that of the hfBP group (88.90%). These results indicated that hfBP increased the risk of blood clotting, showing worse efficacy in anticoagulation (Table 4).

**Table 4.** Filter lifespan and the number of patients with multiple organ dysfunction syndrome treated with regional citrate anticoagulation (RCA) or heparin-free blood purification (hfBP) undergoing filter replacement.

Group	Filter lifespan (h)	Complete replacement (n)	Filter replacement (n)	No replacement (n)	Total (n)	Replacement rate (%)
RCA	29.84±21.34	0	2	15	17	5.90
HfBP	12.46±17.13	15	1	2	18	88.9*
		χ2=20.91	P=0.001			

Data are reported as means±SD.

* P<0.05, hfBP *vs* RCA group (chi-square test).

### Blood clearance

The concentration of creatine (Cr), blood urea nitrogen (BUN) and MODS score were significantly lower after both treatments compared with pre-treatment. Cr and uric acid concentrations in the hfBP group were both significantly higher after treatment than in the RCA group (Table 5). These results indicated that RCA had better clearance outcomes in terms of Cr and uric acid clearance than hfBP.

**Table 5.** Blood clearance parameters and multiple organ dysfunction syndrome (MODS) scores of regional citrate anticoagulation (RCA) and heparin-free blood purification (hfBP) treatment groups.

Variable	RCA group		hfBP group	
	Pre-treatment	Post-treatment	Pre-treatment	Post-treatment
Creatine (µmol/L)	423±462.25	136±104.01*	439.67±216.15	292.72±157.42* Δ
BUN (mmol/L)	25.69±22.08	11.38±10.26*	27.87±15.35	15.91±9.14*
UAC (µmol/L)	338.67±204.91	127.50±75.01	365.94±189.66	214.83±123.58* Δ
MODS score	8.00±3.33	6.33±2.61*	9.39±2.25	8.11±2.89*

Data are reported as means±SD. BUN: blood urea nitrogen; UAC: uric acid.

* P<0.05, post-treatment *vs* pre-treatment;

Δ P<0.05, heparin-free *vs* RCA after treatment. Statistical analysis was performed with the *t*-test.

### Main metabolic and electrolyte indicators

RCA significantly increased systemic pH after treatment, suggesting that RCA could result in metabolic alkalosis during treatment. RCA significantly decreased iCa level after treatment, suggesting that RCA could result in hypocalcemia. Na^+^ and HCO_3_^-^ concentrations had no significant difference before treatment and after treatment in both groups. Similarly, RCA also showed significantly higher pH and lower iCa level than hfBP treatment. Na^+^ and HCO_3_^-^ concentrations had no significant difference between groups (Table 6).

**Table 6.** Main metabolic and electrolyte parameters before and after the treatment of patients with multiple organ dysfunction syndrome treated with regional citrate anticoagulation (RCA) or heparin-free blood purification (hfBP).

Group	pH	iCa (mmol/L)	Na+ (mmol/L)	HCO_3_^-^ (mmol/L)
RCA				
Before treatment	7.35±0.14	1.68±2.26	134.92±5.96	21.32±7.03
After treatment	7.43±0.05* +	1.08±0.10	137.75±3.31	24.16±1.74
hfBP				
Before treatment	7.42±0.09	1.07±0.15	137.67±6.52	21.73±4.38
After treatment	7.44±0.06	1.06±0.14	138.44±4.64	22.69±2.70

Data are reported as means±SD. iCa: ionized Ca.

* P<0.05, before-treatment *vs* after-treatment group;

+ P<0.05, RCA *vs* hfBP group. Statistical analysis was performed with the *t*-test.

### Correlation between coagulation indicators and MODS scores

PT and INR showed no difference among the three MODS score groups. PT activity in the >10–15 group was more intense than the 0-≤5 and >5-≤10 groups. APTT of the >5-≤10 group was significantly lower than in the 0-≤5 and >10–15 groups, and platelet concentration of the >10–15 group was significantly lower than the 0-≤5 and >5-≤10 groups. FIB concentration in the >5-≤10 group was significantly more intense than in the 0-≤5 group. These results indicated a positive correlation between PT activity and MODS score but a negative correlation between platelet concentration and MODS score (Table 7).

**Table 7.** Correlation between multiple organ dysfunction syndrome (MODS) score and coagulation indicators of patients with multiple organ dysfunction syndrome treated with regional citrate anticoagulation or heparin-free blood purification.

MODS score	PT (s)	PT activity (%)	APTT (s)	INR	FIB (g/L)	Platelet (10^9^/L)
0 – ≤5	19.37±2.22	41.70±8.41	41.77±4.54	1.62±0.21	3.59±1.62	130.00±79.89
>5 – ≤10	17.62±2.96	50.45±14.11	33.78±8.77	1.51±0.25	6.23±2.31*	129.19±99.81
>10 – ≤15	16.00±3.02	70.52±41.50* +	61.09±68.26+	1.37±0.32	5.58±1.75	51.51±38.86+

Data are reported as means±SD. PT: prothrombin time; APTT: activated partial thromboplastin time; INR: international normalized ratio; FIB: fibrinogen.

* P<0.05, before-treatment *vs* after-treatment group,

+ P<0.05, RCA *vs* hfBP group. Statistical analysis was performed with one-way ANOVA.

## Discussion

CBP appears to be the last resort to rescue MODS patients due to its advantage of continuity during the treatment. In this process, protecting patients from bleeding is important. However, how to conduct anticoagulation during CBP therapy for MODS patients with a high risk for bleeding still remains problematic (9). We assessed the efficacy and safety of RCA in the CBP treatment in this study. We compared the anticoagulation effects of MODS patients going through RCA or hfBP. By analyzing blood coagulation indicators, blood pressure, blood clearance and the occurrence of complications, we arrived at the conclusion that RCA is a comparatively safe and effective method in CBP treatment of MODS patients.

In our research, we selected ACD-A, a commercially available blood preservation solution, to act as the anticoagulant. There are many indicators to assess anticoagulation efficacy including whole blood activated clotting time, calcium level at the vein side of extracorporeal circulation and the lifespan of filters (16). We chose the lifespan of filters as our primary outcome of efficacy. A longer filter lifespan means a more effective treatment time and less treatment interruption. In our research, a better efficacy of RCA anticoagulation was found. This result was consistent with a published study, in which RCA treatment was related to a longer lifespan of filters compared with the control group (17). Morabito et al. (18) also suggested that RCA could ensure enough filter life and decrease treatment cost.

RCA technology inputs sodium citrate through arterial blood. Citrate and calcium react in the blood and form calcium citrate, which is hard to dissolve, so the activated calcium ions in the blood significantly decrease, thrombin and other blood coagulation processes occur leading to full anticoagulation. The most important aim of hemodialysis is to remove the poisonous solute and metabolites in the patients’ bodies, and it has to be achieved only under the condition of efficient extra-corporeal circuit anticoagulation. Despite the reported efficacy of RCA, several safety endpoints should also be considered. The blood concentration of Cr, BUN, and UAC also significantly decreased after RCA treatment. Compared with the control group, RCA revealed better ability in Cr, BUN and UAC clearance, indicating its higher safety. Citrate cannot be used with hemofiltration alone because of the insufficient clearance of sodium citrate in blood, which could cause the imbalance of serum sodium and bicarbonate concentrations (19). Moreover, high ultrafiltration rate was reported to reach high clearances (19). No report has carefully elaborated the mechanism by which RCA eliminates Cr, BUN and UAC in blood after the blood purification process. However, studies found that citrate anti-coagulation indeed demonstrated better blood solute clearance than other purification methods (20-22). It has been agreed that the excess citrate used in RCA purification can be metabolized via the kidney and liver. We believe that this is related to the good Cr, BUN and UAC clearance results of RCA purification method.

In the present study, we also assessed the blood pressure-related indicators, such as PA, PBE, PV and TMP, to evaluate the safety of different anticoagulants. RCA resulted in lower blood pressure than hfBP, suggesting a lower risk of bleeding. (23). The most common complication of RCA treatment is hypocalcemia, which together with metabolic alkalosis are closely correlated with the use of sodium citrate. In our study, hemorrhage complications were not observed in RCA and hfBP groups; however, it was possible that other complications such as citrate acidosis, hypernatremia and metabolic acid-basic disorder may occur (24). For example, the electrolyte indicator “pH” was higher in patients treated with RCA, suggesting that RCA may result in metabolic alkalosis. However, the risk of metabolic alkalosis when using citrate and bicarbonate is rarely high and could be lower when using a carefully calculated bicarbonate continuous veno-venous hemofiltration algorithm (18,25). Another study pointed out that a lower dialysate bicarbonate concentration (<25 mmol/L) may lower the occurrence of alkalosis (26). As mentioned before, the severest complication caused by citrate infusion is hypocalcemia, which could result in myocardial dysfunction or even death (27,28). The metabolic process of citrate in the body is fast. If we stop the input of sodium citrate, within 10 to 30 minutes the level of citrate and free calcium in the body can be restored to the normal level. Therefore, the proper operation may ensure safety in therapy. For example, metabolic complications could be reduced if a standardized protocol is applied to adjust dialysate flow and calcium substitution so that the blood pH and ionized calcium levels would be maintained within a normal range (29). In the present study, imbalances of iCa were not significantly different between the RCA and hfBP groups after treatment, which is probably because of the small study population. This contradiction requires a further study involving a larger patient cohort to study the ionized calcium imbalance after the treatment of RCA and other anticoagulants.

There are some limitations to this study. First, the sample consisted of only 35 patients, which was limited compared with other studies probing into RCA (30). Second, although this study included a variety of outcomes, it still lacked some important indicators, such as other potential complications like 30-day survival rate (31). Hence, we failed to present an impressive reduction in mortality after the use of RCA. The efficacy and safety of other anticoagulants could also be considered in further studies.

In conclusion, RCA treatment demonstrated safer and more effective outcomes than the hfBP treatment. However, RCA could also lead to several adverse effects such as blood alkalosis and low blood pressure.

## Supplementary material

Click here to view [pdf].
